# The public health impact of extending the catch-up nonavalent human papillomavirus vaccination program to 2028 in Japan: a model-based study

**DOI:** 10.1186/s12889-025-25684-8

**Published:** 2025-12-06

**Authors:** Ibrahim Diakite, Keisuke Tobe, Xuedan You, Ya-Ting Chen, Cody Palmer, Taizo Matsuki

**Affiliations:** 1 Health Economic and Decision Sciences, Merck & Co., Inc., Rahway, NJ USA; 2 MSD K.K., Tokyo, Japan; 3 Outcomes Research, Merck & Co., Inc., Rahway, NJ USA; 4 WP 37A-150, 770 Sumneytown Pike, 1st Floor, West Point, PA 19486 USA

**Keywords:** Dynamic transmission model, Female, Human papillomavirus vaccine, Japan, Male, Vaccination

## Abstract

**Background:**

Human papillomavirus (HPV)-related diseases have risen in Japan since recommendations for the HPV vaccine were suspended in 2013. This study evaluated the public health benefit of extending the current 3-year nonavalent HPV (9vHPV) catch-up vaccination program for females 17–26 years of age to 6 years.

**Methods:**

A dynamic transmission model calibrated to Japanese specific demographic and epidemiological data compared the impact of different 9vHPV vaccination scenarios on female and male HPV-related diseases over a 100-year time horizon. The base-case analysis compared the routine vaccination in combination with the current 3-year catch-up program for females 17–26 years of age from 2022 to 2025 (status quo) to the routine vaccination in combination with an extended 6-year program from 2022 to 2028 (6-year catch-up scenario) for Japan.

**Results:**

HPV-related disease incidence with the 6-year catch-up scenario decreased further than the status quo over 100 years. Extending catch-up vaccinations from three to six years in combination with routine vaccination prevented an additional 413,454 HPV-related disease cases and 7,184 deaths for both sexes. Anogenital warts had the highest number of averted cases (361,020 cases) and males (229,867 cases) had more total HPV-related disease cases averted than females (183,586 cases).

**Conclusions:**

Routine vaccination in combination with an extension of the catch-up HPV vaccination strategy for females 17–26 years of age from three to six years may expedite the decrease of morbidity and mortality due to HPV-related diseases in Japan.

## Plain language summary

The vaccine-preventable human papillomavirus (HPV) infects most sexually active individuals during their lifetime and high coverage rates of the prophylactic HPV vaccines has been shown to significantly lower the incidence of HPV-related diseases. Cervical cancer is the most common HPV-related cancer, and it is the second leading cause of cancer-related deaths in Japan. HPV vaccinations were introduced in Japan in 2010, and almost three quarters of females 13–16 years of age were fully vaccinated by April 2013. Despite the favorable safety data for HPV vaccines worldwide, Japanese health officials suspended recommendations for HPV vaccinations in 2013 after incorrect information spread regarding harmful side effects from the vaccine. After nine years and rises in HPV-related disease incidence, officials reinstated proactive HPV vaccine recommendations for females 12–16 years of age along with a three-year catch-up vaccination program for females 17–26 years of age. This study used a dynamic transmission model for HPV to determine the impact of extending the current three-year catch-up HPV vaccination program (2022–2025) to six years (2022–2028) over a 100-year time frame in Japan. The results indicate that extending catch-up vaccinations an additional three years may prevent an additional 413,454 HPV-related disease cases and 7,184 deaths for both sexes. Interestingly, there were 1.3 times as many total HPV-related disease cases averted for males than females due to a greater amount of male anogenital wart cases averted (225,435 male cases vs. 135,585 female cases), demonstrating the indirect benefit of extending the female catch-up program on HPV-related diseases in males. This study demonstrates that an extension of the catch-up HPV vaccination strategy for females 17–26 years of age from three to six years may improve HPV-related disease burden in Japan.

##  Background

The vaccine-preventable human papillomavirus (HPV) infects most sexually active individuals during their lifetime [1]. Persistent HPV infection may lead to a variety of long-term disease conditions including anogenital and oropharyngeal cancers, anogenital warts (AGWs), and recurrent respiratory papillomatosis [2–4]. Cervical cancer is the most common HPV-related cancer worldwide with an average age-standardized rate (ASR) of 13.3 cases per 100,000. The ASR of cervical cancer in Japan has steadily been increasing over the last twenty years from 9.1 to 15.3 cases per 100,000 (2000–2020) [5, 6]. The current ASR of cervical cancer in Japan (15.3 cases per 100,000) is much higher than in other developed nations such as the United States (6.5 cases per 100,000) and South Korea (8.4 cases per 100,000), and cervical cancer is the second leading cause of all cancer-related mortality in Japan, leading to 4,200 deaths annually [5, 7, 8].

Most HPV-related diseases have no early detection or treatment options; therefore, vaccination prior to the start of sexual activity is crucial for preventing an initial HPV infection that may lead to future disease [4]. All HPV vaccinations have had favorable safety data as effective preventive measures for HPV infection [1, 9, 10]. The nonavalent HPV vaccine (9vHPV), effective against nine HPV genotypes (6, 11, 16, 18, 31, 33, 45, 52, and 58), has the potential to prevent 21% of oropharynx, 23% of vulvar, 25% of penile, 61% of vaginal, 79% of anal, 95% of cervical cancers [4, 11]. Additionally, 9vHPV can prevent 85–90% of recurrent respiratory papillomatosis and AGW cases for both males and females [4, 12, 13]. A meta-analysis including 65 publications from fourteen high-income countries (including Australia, New Zealand as well as European and North American countries) compared the incidence of HPV-related diseases before vaccination to over eight years of post-HPV vaccination follow-up [14]. The results indicated a strong correlation between HPV vaccination coverage and a lower incidence of HPV-related diseases, including cervical precancers such as cervical intraepithelial neoplasia (CIN) [14].

A 3-dose vaccine series of either the HPV bivalent vaccine (2vHPV) or quadrivalent vaccine (4vHPV) has been subsidized for females 12–16 years of age in Japan since 2010 [4, 15, 16]. By April 2013, full vaccination coverage of females 13–16 years of age was 70%, and the HPV vaccine was added to the national immunization program (NIP) at no cost to parents [17–19]. However, the Japanese Ministry of Health, Labor, and Welfare (MHLW) suspended proactive recommendations for the vaccine in June 2013 after incorrect information spread in the media regarding adverse health events after HPV vaccinations [20]. Despite the availability of the HPV vaccine within Japan’s NIP, confusion regarding the safety and benefit of the vaccine led to less than 1% HPV vaccine coverage for 13-year-old females in 2013 [18, 20]. Unfortunately, the impact of lower HPV vaccine coverage is already evident with the higher occurrence of abnormal cervical cytology for Japan’s year 2000 birth cohort [21]. Simms et al. used a dynamic model of HPV transmission to look at the long-term consequences of the drop in HPV vaccination coverage in Japan [19]. If a 70% HPV vaccination coverage rate had been maintained in Japan since 2013, 24,600−27,300 cases and 5,000–5,700 deaths due to cervical cancer would have been prevented for the 1994–2007 birth cohort over their lifetimes [19]. Modeling studies have not only indicated the importance of increasing routine coverage back to 70% levels, but also adding catch-up vaccination programs for females 17–26 years of age to lower long-term HPV-related disease incidence and deaths even further in Japan [19, 22].

In November 2021, the MHLW reinstated its proactive recommendation of routine HPV vaccinations for females 12–16 years of age [23, 24]. Additionally, it began to fund a catch-up HPV vaccination program from 2022 to 2025 for unvaccinated female birth cohorts from 1997 to 2006 (females 17–26 years old) [2, 25]. The three-year catch-up period was established as a relief measure specifically designed to provide vaccination opportunities to individuals who were unable to receive the HPV vaccine during the period of recommendation suspension (2013–2022) [26]. When the 9vHPV vaccine was added to the NIP in 2023, the MHLW revised its recommendations to two doses of 9vHPV for females 12–14 years of age and three doses of 9vHPV for females >15 years of age [27]. Current estimates indicate that the single HPV vaccine dose coverage for females 12–16 years of age in 2022 was only 14.2% while single-dose catch-up vaccination coverage was 12.0% and 21.2% for birth cohorts from 2000 to 2005 and 1997–1999, respectively [8]. These vaccination coverage levels are much lower than those desired to help mitigate future HPV-related disease burden in Japan.

Japan’s MHLW’s return to recommending the HPV vaccine for both routine and catch-up vaccinations is a critical step toward eliminating HPV as a public health concern in Japan. However, the continual low routine and catch-up HPV vaccination rates indicate concerns remain in Japan regarding the safety and effectiveness of the vaccine. The objective of this study was to determine the public health benefit of extending the current 9vHPV catch-up vaccination program for females 17–26 years of age to a total of six years from 2022 to 2028, similar to the previous extension period observed for the pneumococcal vaccination program in Japan [28]. The results from the study may help guide Japan’s public health decisions to lower morbidity and mortality due to HPV-related diseases.

##  Methods

### Study overview

An established dynamic transmission model was adapted to determine the public health impact of routine vaccination in combination with extended catch-up program to six years (6-year catch-up scenario) for females 17–26 years of age in Japan compared to routine vaccination in combination with three years catch-up program (status quo) over a 100-year time horizon (2020–2120) [22, 27, 29, 30]. Public health outcomes are reported as HPV-related disease incidence over time, as well as cumulative averted cases and deaths due to HPV-related conditions (precancers, cancers, and other diseases).

### Model

The dynamic transmission model for this analysis was adapted from previous 9vHPV modeling studies for Japan [22, 27]. The inputs and parameters included Japan-specific demographics, sexual behavior, routine HPV vaccination rates, cervical cancer screening and treatment rates, 9vHPV strain prevalence, HPV-related disease incidence, rate of progression to CIN1, 2, or 3, and other HPV transmission dynamics described in more detail in previous studies [22, 27]. Following the timeline of approval in Japan, the 4vHPV vaccine was input into the model for 2022 and the 9vHPV vaccine was inputted for 2023 and beyond. The model outputs included the incidence of HPV-related cancers and other diseases over time (cervical, non-cervical, AGW, and juvenile-onset recurrent respiratory papillomatosis (JORRP)) as well as specific cases of HPV-related conditions and deaths averted (CIN, cervical, vaginal, and vulvar cancers in women; penile cancer in men; and anal cancer, oropharyngeal cancer, AGW, and JORRP in men and women) during a 100-year time horizon (2020–2120). Estimated HPV-related cases and deaths in this study specifically refer to HPV-related cases and deaths due to HPV types found in the 9vHPV vaccine. The total HPV-related conditions and deaths averted were calculated by subtracting the estimated number of cases or deaths from the status quo scenario by the estimated number of cases or deaths for the 6-year catch-up scenarios at year 100.

### Modification to published model inputs

For this study, the input routine and catch-up female HPV vaccination coverage rates began in 2022, the year MHLW reinstated its recommendation of the HPV vaccine for routine and catch-up vaccinations. Routine coverage was input as 10% for females aged 12–16 years of age in 2022, increasing by 10% each year until 30% in 2025, and remaining at 30% for the rest of the model’s time horizon for all scenarios. The catch-up vaccination coverage for females 17–26 years of age was input as 10% in 2022, increasing by 10% each year until 30% in 2025 for the status quo. For the 6-year catch-up, the catch-up coverage further increased to 40% in 2026, 50% in 2027, and 65% in 2028 (Table 1). Baseline historical catch-up vaccination coverage of females 17–26 years of age was input as 28.81% and 29.92% for 2020 and 2021, respectively. These values were calculated with catch-up vaccination data for individuals with birth years between 1997 and 2005 from the Japan Medical Data Survey database (JAMDAS), an outpatient health care database of nationwide primary care physician clinics in Japan [32]. The levels of routine and catch-up vaccination coverage for the base analysis were selected to be representative of current and anticipated coverage levels in Japan from published MHLW statistical data, current and projected vaccine sales, and discussions with local public health experts [20, 27, 33, 34].

**Table 1:** Catch-up and routine VCR for the base-case and sensitivity analysis scenarios for females in Japan ^A^

Scenarios		Catch-up 9vHPV VCR								
	Year 1	Year 2	Year 3	Year 4	Year 5	Year 6	Year 7+			
Base-case analysis ^B^										
* Status quo (3-year catch-up)*										
Routine VCR ^C^	10%	20%	30%	30%	30%	30%	30%			
Catch-up VCR	10%	20%	30%	0%	0%	0%	0%			
* 6-year catch-up*										
Routine VCR ^C^	10%	20%	30%	30%	30%	30%	30%			
Catch-up VCR	10%	20%	30%	40%	50%	65%	0%			
Sensitivity analysis (alternative catch-up) ^D^										
6-year catch-up-LC	10%	20%	30%	35%	40%	50%	0%			
6-year catch-up-HC	10%	20%	30%	45%	60%	80%	0%			
		**Routine 9vHPV VCR**								
	**Year 1**	**Year 2**	**Year 3**	**Year 4**	**Year 5**	**Year 6**	**Year 7**	**Year 8**	**Year 9**	**Year 10+**
Sensitivity analysis (alternative routine VCR) ^E^										
Routine VCR-60% ^F^	10%	14%	18%	23%	29%	36%	44%	52%	60%	60%
Routine VCR- 80% ^F^	10%	15%	21%	28%	37%	46%	57%	68%	80%	80%
Routine VCR-90% ^F^	10%	16%	23%	31%	40%	51%	63%	76%	90%	90%

*9vHPV* nonavalent human papillomavirus vaccine, *LC* lower coverage, *HC* higher coverage, *VCR* vaccine coverage rate

^A^ Each year refers to a fiscal year starting April 1. For instance, year 1 starts April 1, 2022, and ends March 31, 2023, while year 6 starts April 1, 2027 and ends March 31, 2028. The base-case and alternative catch-up sensitivity analysis scenarios include a routine VCR of 10% for females 12–16 years of age in 2022 (year 1) that increases to a fixed level of 30% for 2025 (year 3) and beyond

^B ^The base-case analysis of this study compares the routine vaccination + 3-year catch-up scenario (status quo) with routine vaccination + an extended 6-year catch-up scenario

^C^ The routine VCR is maintained at 30% for the rest of the 100-year time horizon

^D^ The alternative catch-up sensitivity analysis compares higher and lower catch-up vaccination coverage rates for the 6-year catch-up scenario vs. the status quo scenario

^E^ The alternative routine VCR sensitivity analysis compares routine vaccination + 3-year catch-up and routine vaccination + 6-year catch-up scenarios in the context of increases in routine VCR for females 12–16 over nine years. For instance, the routine + 3-year catch-up scenario was compared to the routine vaccination + 6-year catch-up scenario with a routine VCR increasing to reach 60% by year 9. The WHO has called for increases in cervical cancer screening, treatments and HPV vaccinations (90% coverage) by 2030 (year 9) to help eliminate cervical cancer (< 4 cases/100,000 women) globally [31]

^F^ The routine VCR is maintained at the year-9 coverage level (60%, 80%, or 90%) for the rest of the 100-year time horizon

### Scenario analysis

In the base-case analysis, the status quo was compared to the 6-year catch-up scenario (Table 1). Year 1 was from April 2022 to March 2023, while year 6 was from April 2027 to March 2028. The years ran from April 1 to March 31, based on the fiscal year of the Japanese government. The 6-year catch-up 9vHPV coverage rates were the same as the status quo for years 1 to 3, while 9vHPV coverage rates were 40%, 50% and 65% for years 4 to 6 (Table 1). Two separate sensitivity analyses were run to evaluate assumptions from the model. One analysis compared two additional 6-year catch-up scenarios to the status quo in the base-case to determine the impact of higher or lower 9vHPV catch-up coverage rates (in the extended three-year period) on HPV-related public health outcomes. The lower coverage scenario (6-year catch-up-LC) had coverage rates of 35%, 40% and 50% for years 4, 5, and 6, respectively. The higher coverage scenario (6-year catch-up-HC) had coverage rates of 45%, 60% and 80% for years 4, 5, and 6, respectively (Table 1). These alternative catch-up vaccination coverage rates were selected after discussions with local public health experts. The second sensitivity analysis evaluated differences in public health outcomes between the routine vaccination + 3-year and routine vaccination + 6-year HPV catch-up programs in the context of increases in routine vaccine coverage rates (VCR) from 30% in 2022 to 60%, 80%, or 90% by 2030 (Table 1). For instance, a routine vaccination + 3-year catch-up program was compared to routine vaccination + 6-year catch-up program under the assumption that routine vaccination VCR would increase to reach 60% by 2030, etc. The highest increase in VCR aligns with the World Health Organization (WHO) global strategy to eliminate cervical cancer by increasing routine VCR for females to 90% worldwide by 2030 [31]. For all scenarios in the study, it was assumed that from April 2023 onwards, females < 15 years of age would receive two doses of 9vHPV, while those >15 years of age received three doses of 9vHPV. We assumed that series completion for those below 15 years was two doses; thus, the vaccine efficacy estimates for 2-dose (for < 15 years) and 3-dose (for ≥ 15 years) were assumed to be the same. Disease and serotype-specific vaccine efficacy assumptions are fully described in our previous publications of the model [22, 27].

##  Results

### Base-case analysis

**Fig. 1:**
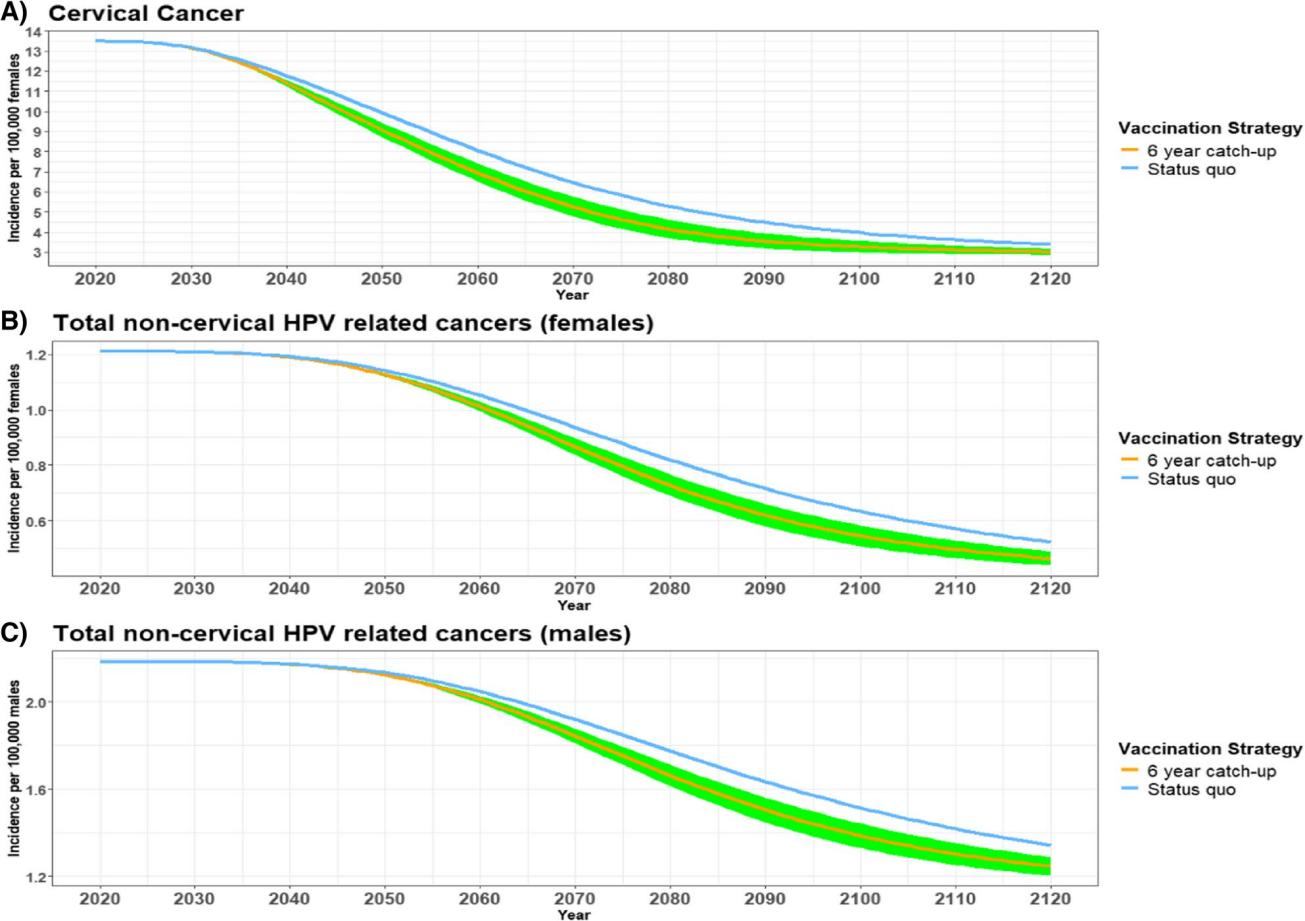
Estimated HPV-related cancer incidence in Japan from 2020–2120. The estimated incidence of (**A**) cervical cancer, (**B**) total non-cervical HPV-related cancers (female), and (**C**) total HPV-related cancers (male) with routine vaccination + either a 3-year (status quo) or 6-year catch-up HPV vaccination program for females 17–26 years of age. The shaded green area represents the incidence estimates from the lower and higher catch-up vaccination coverage scenarios from the sensitivity analysis (Table 1)

Annual 9vHPV-related cervical cancer incidence declined more rapidly and further with the 6-year catch-up scenario than the status quo (Fig. 1A). Cervical cancer cases declined from 13.48/100,000 in 2020 to 3.38/100,000 and 3.01/100,000 by 2120 for the routine vaccination plus 3-year catch-up (status quo) and 6-year catch-up, respectively (Fig. 1A). Although this analysis only accounted for vaccine-preventable HPV types, the results indicate that routine vaccination in combination with the 6-year extension is likely to accelerate cervical cancer elimination by roughly 20 years compared to the status quo (Fig. 1A). Total non-cervical 9vHPV-related cancers in females declined from 1.21 cases/100,000 in 2020 to 0.52 cases/100,000 and 0.46 cases/100,000 with the status quo and 6-year catch-up scenario, respectively by 2120 (Fig. 1B). A similar trend occurred for total HPV-related cancers in males where 2.18 cases/100,000 in 2020 declined to 1.34 cases/100,000 and 1.25 cases/100,000 with the status quo and 6-year catch-up, respectively by the end of the model’s time horizon (Fig. 1C).

**Fig. 2:**
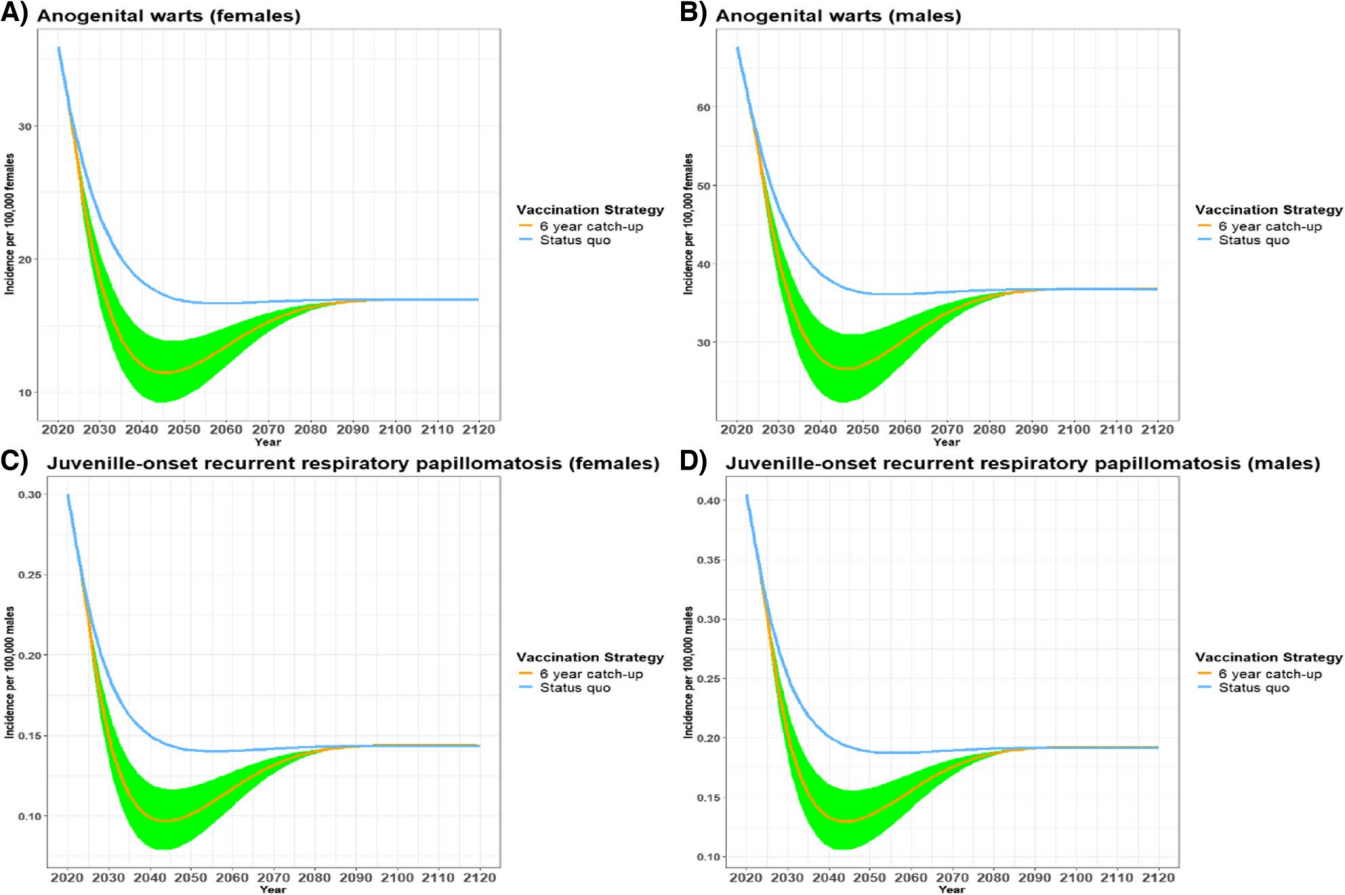
Estimated non-cancerous HPV-related disease incidence in Japan from 2020–2120. The estimated incidence of (**A**) Anogenital warts (females), (**B**) Anogenital warts (males), (**C**) Juvenile-onset recurrent respiratory papillomatosis (females), and (**D**) Juvenile-onset recurrent respiratory papillomatosis (males) with routine vaccination + either a 3-year (status quo) or 6-year catch-up HPV vaccination program for females 17–26 years of age. The shaded green area represents the incidence estimates from the lower and higher catch-up vaccination coverage scenarios from the sensitivity analysis (Table 1)

The 6-year catch-up scenarios for both male and female AGW and JORRP cases showed a steeper initial decline in cases than the status quo followed by an increase back to similar steady state incidence levels as the status quo by the end of the horizon (Fig. 2). For example, AGW female cases declined further and faster from 35.95 cases/100,000 in 2020 to 11.46 cases/100,000 by 2045 for the 6-year catch-up scenario compared with 16.67 cases/100,000 in 2058 for the status quo. Female AGW cases then gradually increased and plateaued at 16.90 cases/100,000 by 2092 for the remainder of the horizon for both scenarios (Fig. 2A) as the catch-up programs end after the extension period. For males, AGW cases declined from 67.69 cases/100,000 in 2020 to 26.59 cases/100,000 by 2046 for the 6-year catch-up compared with 36.1 cases/100,000 in 2057 for the status quo. Cases then gradually increased and plateaued at 36.70 cases/100,000 by 2093 for the remainder of the horizon for both scenarios (Fig. 2B). JORRP cases showed a similar trend as AGW cases with the 6-year catch-up. JORRP female cases declined from 0.30 cases/100,000 in 2020 to 0.10 cases/100,000 by 2045 for the 6-year catch-up compared with 0.14 cases/100,000 by 2056 for the status quo (Fig. 2C). For males, JORRP cases declined from 0.40 cases/100,000 by 2020 to 0.13 cases/100,000 by 2044 for the 6-year catch-up compared with 0.19 cases/100,000 by 2056 for the status quo (Fig. 2D). JORRP cases for both base-case scenarios are estimated to increase and plateau at 0.14 cases/100,000 for females by 2074 and 0.19 cases/100,000 for males by 2078 for the remainder of the horizon (Fig. 2C &D).

In the base-case analysis, routine vaccination with an extension of female catch-up vaccinations from three to six years was expected to avert an additional 413,454 cases and 7,184 deaths due to HPV-related cancers and diseases for both sexes over 100 years (Table 2). Total HPV-related cancer and other disease cases averted were higher in males than females (229,867 males, 183,586 females), mostly due to a greater number of averted AGW cases for males (225,435 cases) than females (135,585 cases). Females had a higher number of averted cancer cases and deaths than males (females: 47,803 cases and 5,814 deaths; males: 4,147 cases and 1,347 deaths), primarily due to cervical cancer cases (44,589 cases and 5,033 deaths). The analysis also predicted that 274,665 cervical precancerous lesions (CIN1, 2, and 3) would be averted with the extended catch-up program. Total cancer cases and deaths averted in males were mostly due to oropharyngeal cancers (3,132 cases and 1,201 deaths; Table 2).

**Table 2:** Total averted cancer and disease cases due to routine vaccination of HPV + 6-year catch-up vs. 3-year catch-up (status quo) program over 100 years in Japan ^A^

	Female				Male					Total			
	Cases	Deaths	Cases PF (%)	Deaths PF (%)	Cases	Deaths		Cases PF (%)	Deaths PF (%)	Cases	Deaths	Cases PF (%)	Deaths PF (%)
Precancerous lesion													
CIN1	56,390	N/A	9.0	N/A	N/A	N/A	N/A		N/A	56,390	N/A	9.0	N/A
CIN2-3	218,275	N/A	9.2	N/A	N/A	N/A	N/A		N/A	218,275	N/A	9.2	N/A
Cancer													
Cervical	44,589	5,033	10.6	11.4	N/A	N/A	N/A		N/A	44,589	5,033	10.6	11.4
Anal	1,219	157	14.9	15.3	965	137	16.0		16.3	2,184	294	15.4	15.7
Oropharyngeal	1,083	383	15.1	15.9	3,132	1,201	16.9		17.5	4,215	1,584	16.4	17.1
Vaginal	579	182	14.1	14.9	N/A	N/A	N/A		N/A	579	182	14.1	14.9
Vulvar	333	59	13.3	13.9	N/A	N/A	N/A		N/A	333	59	13.3	13.9
Penile	N/A	N/A	N/A	N/A	50	9	16.0		17.0	50	9	16.0	17.0
Total	47,803	5,814	10.8	11.8	4,147	1,347	16.7		17.4	51,951 ^B^	7,161	11.1	12.5
Other diseases													
AGW	135,585	N/A	8.5	N/A	225,435	N/A	9.7		N/A	361,020	N/A	9.2	N/A
JORRP	198	9	8.4	8.8	285	14	8.4		8.8	483	23	8.4	8.8
Total cancer and other diseases	183,586	5,823	9.0	11.8	229,867	1,361	9.8		17.2	413,454 ^B^	7,184	9.4	12.5

*AGW* anogenital warts, *CIN* cervical intraepithelial neoplasia, *HPV* human papillomavirus, *JORRP* juvenile-onset recurrent respiratory papillomatosis,* N/A* not applicable, *PF* prevented fraction

^A^ The health impact of the routine HPV vaccination + 6-year-catch-up vaccination program includes the number of 9vHPV-related disease cases and deaths averted over 100 years in Japan. The total estimated cases and deaths averted for each HPV-related condition was calculated by subtracting the total number of cases or deaths for the routine HPV vaccination + 3-year catch-up scenario (status quo) by the total number of cases or deaths for the routine HPV vaccination + 6-year catch-up scenario after 100 years

^B^ Difference in total number is due to rounding of data

### Sensitivity analysis

The first sensitivity analysis looked at the impact of lower (6-year catch-up-LC) and higher (6-year catch-up-HC) catch-up vaccination rates on HPV-related cases and deaths averted over 100 years (Table 3; Figs. 1 and 2). Compared to routine vaccination + 3-year catch-up (status quo), the routine vaccination + 6-year catch-up-HC had the greatest number of disease cases (including AGW, JORRP, and cancer) and deaths averted over 100 years (Table 3). Increasing total catch-up coverage to 80% by year 6 with the 6-year catch-up-HC would avert 42% more 9vHPV-related disease cases (589,117 vs. 413,454 total disease cases for the 6-year catch-up), while the 6-year catch-up-LC with only 50% catch-up coverage by year 6 would avert 43% fewer 9vHPV-related disease cases (233,713 total disease cases). Additionally, 41% more deaths would be averted for both sexes with the 6-year catch-up-HC strategy compared to 43% fewer deaths with the 6-year catch-up-LC strategy over 100 years (Table 3).

**Table 3:** Total averted cancer and disease cases due to routine vaccination with alternative six-year HPV catch-up scenarios vs. routine vaccination with three-year catch-up over 100 years in Japan ^A^

Averted total cases compared to routine vaccination + 3-year catch-up	CIN cases	All cancer cases			AGW cases			JORRP cases			Deaths		
	Total	Female	Male	Total	Female	Male	Total	Female	Male	Total	Female	Male	Total
Routine vaccination + 6-year catch-up	274,665	47,803	4,147	51,951 ^B^	135,585	225,435	361,020	198	285	483	5,823	1,361	7,184
Routine vaccination + 6-year catch-up-LC	158,417	27,518	2,319	29,837	77,338	126,263	203,601	113	163	275 ^B^	3,351	760	4,111
Routine vaccination + 6-year catch-up-HC	383,143	66,816	5,969	72,785	191,357	324,293	515,650	279	403	682	8,139	1,962	10,101

*AGW* anogenital warts, *CIN* cervical intraepithelial neoplasia, *HC* higher coverage, *HPV* human papillomavirus, *JORRP* juvenile-onset recurrent respiratory papillomatosis, *LC* lower coverage

^A^ The health impact is the number of 9vHPV-related disease cases and deaths averted over 100 years in Japan for routine vaccination with each alternative 6-year catch-up scenario compared to the routine vaccination + 3-year catch-up scenario. Total cases and deaths averted were calculated by subtracting the total estimated number of cases or deaths for the routine vaccination + 3-year catch-up scenario (status quo) by the total number of cases or deaths estimated for routine vaccination + each of the 6-year catch-up scenarios after 100 years

^B^ Difference in total number is due to rounding of data

**Fig. 3:**
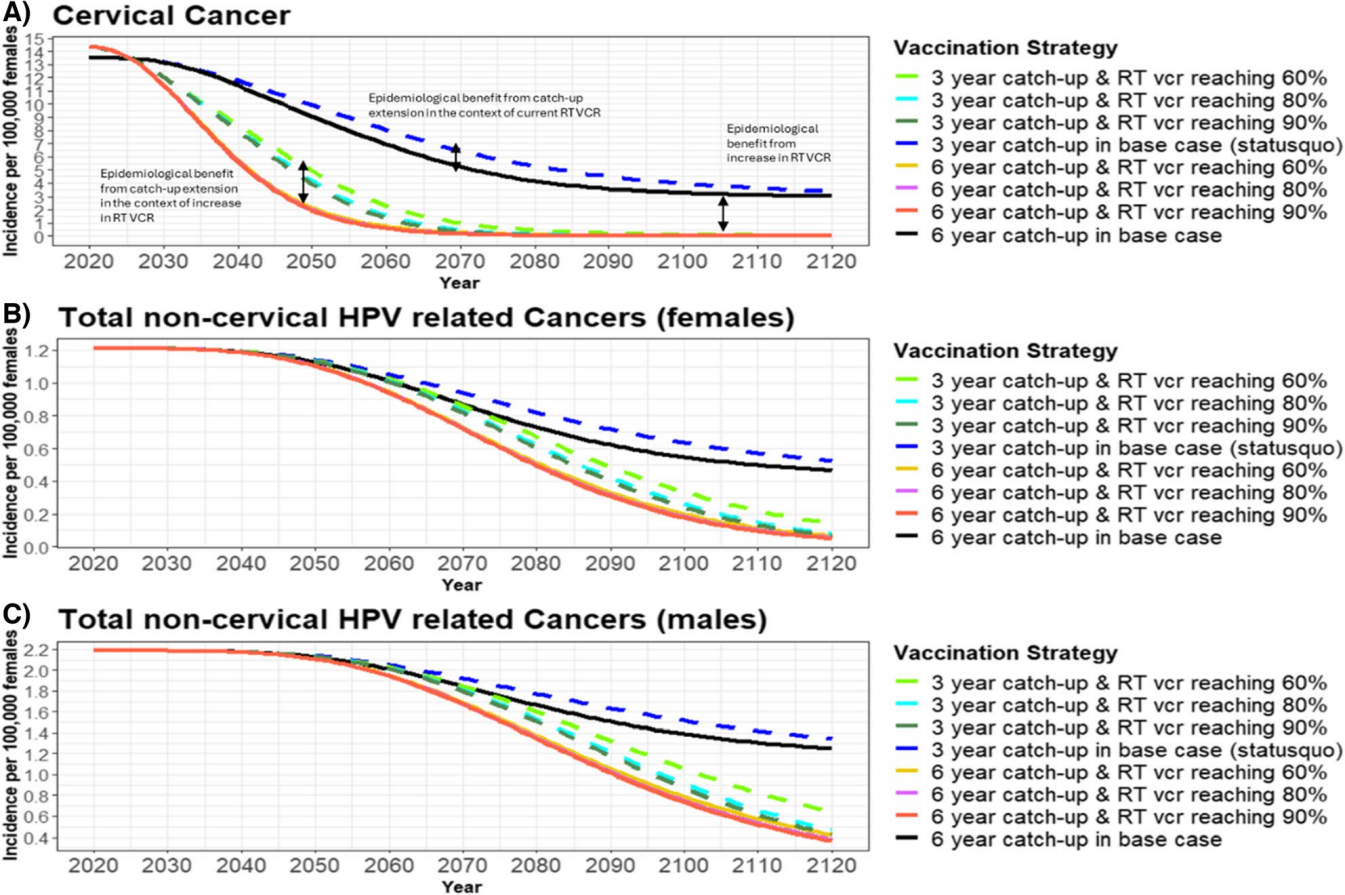
Estimated HPV-related cancer incidence for three and six-year HPV catch-up program with alternative routine HPV VCR in Japan from 2020–2120. RT, routine; VCR, vaccination coverage rate. The estimated incidence of (**A**) cervical cancer, (**B**) total non-cervical HPV-related cancers (female), and (**C**) total HPV-related cancers (male) with routine vaccination + either a 3-year (status quo) or 6-year catch-up HPV vaccination program for females 17–26 years of age and alternative routine VCR (Table 1)

**Fig. 4:**
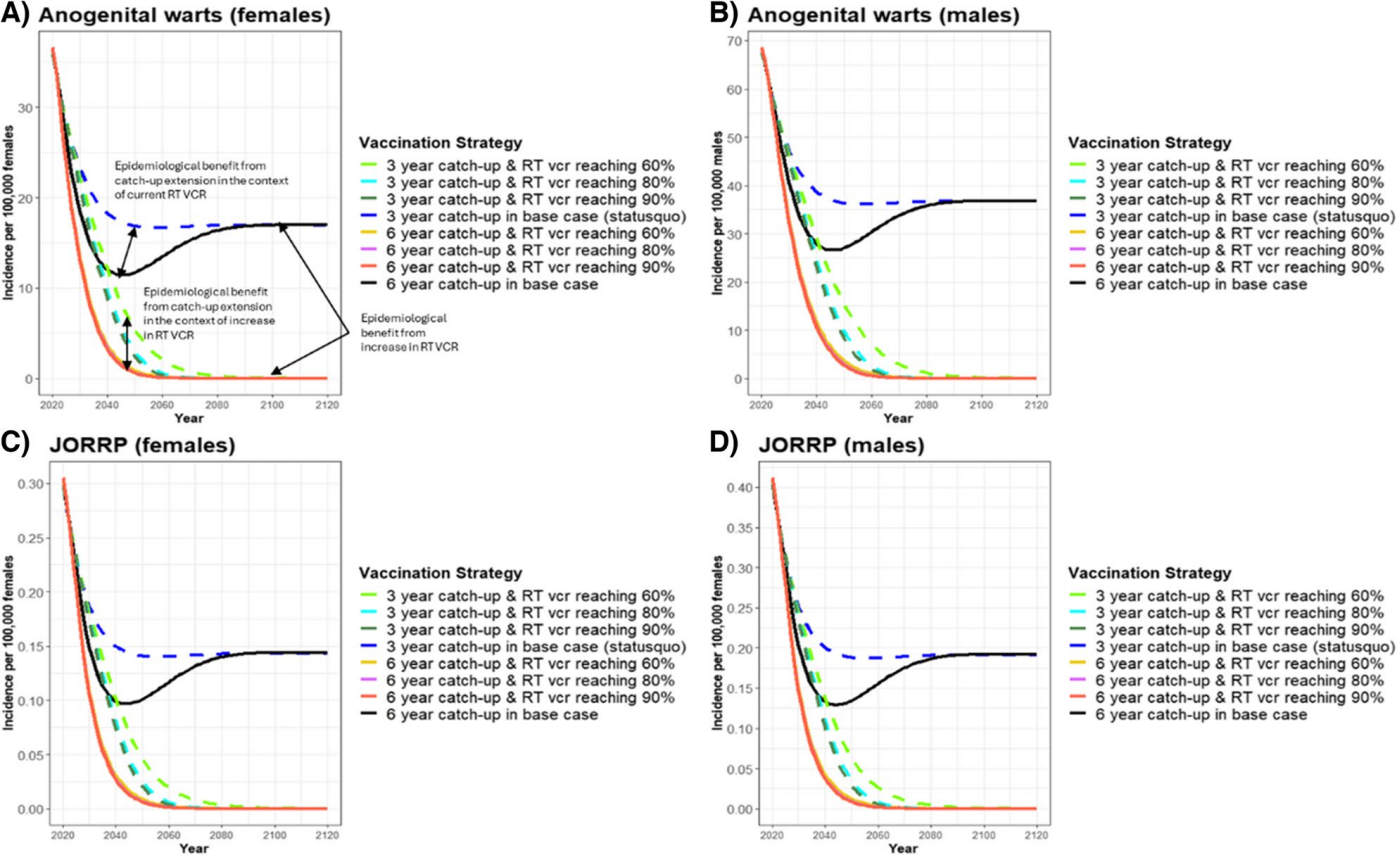
Estimated non-cancerous HPV-related disease incidence for three and six-year HPV catch-up program with alternative routine HPV VCR in Japan from 2020–2120. JORRP, juvenile-onset recurrent respiratory papillomatosis; RT, routine; VCR, vaccination coverage rate. The estimated incidence of (**A**) Anogenital warts (females), (**B**) Anogenital warts (males), (**C**) Juvenile-onset recurrent respiratory papillomatosis (females), and (**D**) Juvenile-onset recurrent respiratory papillomatosis (males) with routine vaccination + either a 3-year (status quo) or 6-year catch-up HPV vaccination program for females 17–26 years of age and alternative routine VCR (Table 1)

The second sensitivity analysis compared routine vaccination + 3-year catch-up vs. routine vaccination + 6-year catch-up under the scenarios where routine VCR in Japan would increase to 60%, 80% or 90% by 2030 (Table 4; Figs. 3 and 4). Compared to routine vaccination + 3-year catch-up, increasing the routine VCR to 60% by 2030 resulted in routine vaccination + 6-year catch-up to avert more total disease cases (562,907) and deaths (10,456) than any other increase in routine VCR evaluated (Table 4). An increase to 60% routine VCR had routine vaccination + 6-year catch-up estimated to prevent an additional 36% 9vHPV-related disease cases and 46% more deaths than the routine vaccination + 3-year catch-up program in the base-case analysis (i.e., when only 30% routine VCR was achieved) (Table 4). However, when comparing routine vaccination + 3-year catch-up vs. routine vaccination + 6-year catch-up, further increases of routine VCR to 80% and 90% by 2030 had similar or lower levels of 9vHPV-related disease cases and deaths averted as the base-case analysis with only a 30% routine VCR. This corresponds to an estimated overall lower incidence of 9vHPV-related diseases in Japan for both sexes due to increases in both routine VCR and extended catch-up vaccinations over 100 years (Table 4; Figs. 3 and 4). For example, cervical cancer incidence was predicted to reach the WHO threshold for the elimination of cervical cancer (4 cases/100,000) by 2080 with the base-case 6-year catch-up extension scenario (30% routine VCR), compared to 2044 for each alternative 6-year catch-up scenario with increases in routine VCR to 60%, 80%, or 90% (Fig. 3) [31]. However, if there was no extension of the current catch-up program, an increase in routine VCR from 60% to 90% would not reach the WHO elimination threshold until 2050 or 2055 (Fig. 3) [31]. For AGW and JORPP, a 6-year catch-up extension (with routine VCRs 60–90%) would also accelerate the reduction of disease cases for both sexes compared to the 3-year catch-up program (routine VCRs 60–90%) (Table 4; Fig. 4).

**Table 4:** Total averted cancer and disease cases due to routine vaccination with six-year HPV catch-up scenarios vs. routine vaccination with three-year catch-up in the context of increasing routine VCR over 100 years in Japan ^A^

Analysis ^B^	CIN	Cancer cases			AGW cases			JORPP cases			Deaths		
	Total	Female	Male	Total	Female	Male	Total	Female	Male	Total	Female	Male	Total
6-year catch-up vs. 3-year catch-up													
Routine VCR reaching 30% (Base-case)	274,665	47,803	4,147	51,951	135,585	225,435	361,020	198	285	483	5,823	1,361	7,184
Routine VCR reaching 60%	303,985	58,344	8,860	67,204	159,911	335,167	495,079	256	368	624	7,482	2,973	10,456
Routine VCR reaching 80%	217,458	41,131	5,962	47,093	113,075	225,437	338,511	177	255	431	5,324	2,036	7,360
Routine VCR reaching 90%	196,283	36,728	5,153	41,880	101,961	201,015	302,976	159	228	387	4,760	1,769	6,529

*AGW* anogenital warts, *CIN* cervical intraepithelial neoplasia, *HC* higher coverage, *HPV *human papillomavirus, *JORRP* juvenile-onset recurrent respiratory papillomatosis, *LC* lower coverage, *VCR* vaccine coverage rate

^A^ The health impact of alternative routine VCRs is the number of 9vHPV-related disease cases and deaths averted for the 3-year and 6-year catch-up scenarios with the same increase in routine VCR (60%, 80% or 90%) by year 9 over 100 years in Japan (Table 1). Total cases and deaths averted for each alternative routine VCR scenario were calculated as the difference in cases and deaths between a 3-year and 6-year catch-up scenarios with the same alternative routine VCR (60%, 80% or 90%) after 100 years

^B^ Base-case analysis refers to the comparison of the 6-year catch-up and 3-year catch-up program, both with a 30% routine VCR after three years. Routine VCR-60%, Routine VCR-80%, and Routine VCR-90% refer to the comparison of the 6-year catch-up and 3-year catch-up programs, each with the same increase in routine VCR

##  Discussion

The model estimates that an additional 413,454 and 7,184 HPV-related cancer and other disease cases and deaths for both sexes will be averted in Japan over 100 years, if routine vaccination implemented with the current HPV vaccine catch-up program is extended for an additional three years. There were 1.3 times as many total HPV-related disease cases averted for males than females due to a greater number of male AGW cases averted (225,435 male cases vs. 135,585 female cases). This demonstrates the indirect benefit of extending the female catch-up program on HPV-related diseases in males. For females, cervical cancer incidence declined further with the routine vaccination + 6-year catch-up than the status quo (routine vaccination + 3-year catch-up), reaching 3.10 cases/100,000 by 2120. Although this study focused on cervical cancers due to 9vHPV vaccine-preventable types, the results suggest that Japan will be able to reach cervical cancer elimination (defined by the WHO as < 4 cases/100,000 women) almost 20 years earlier with the routine vaccination in combination with the 6-year catch-up program than the status quo [31]. The status quo reached elimination by 2100, while the 6-year catch-up reached elimination by 2082.

Despite the proven effectiveness of HPV vaccinations to prevent future HPV infections, if individuals have a persistent HPV infection, precancerous cervical lesions, or genital warts at the time of vaccination, the vaccines are unable to accelerate clearance of that infection [35–37]. Routine HPV vaccines are generally given to children 11 and 12 years of age due to epidemiological data on the age of sexual debut and higher antibody titers for this age group after vaccination [12, 37]. However, older adolescents and young adults through the age of 26 who missed the opportunity for earlier vaccination may still benefit from a delayed vaccination [12, 36, 37]. Short-term HPV catch-up vaccinations for older cohorts in Japan have previously been shown to be a cost-effective tool for reducing the incidence of HPV-related diseases and deaths, especially when routine vaccination and cervical cancer screenings are at suboptimal levels [19, 22].

HPV vaccines are a key prophylactic measure to prevent HPV-related diseases since many HPV infections are difficult to detect and treat prior to the occurrence of symptoms [38]. For instance, AGW cases may appear two to three months after HPV infection, are frequently asymptomatic, and treatment may not eliminate the risk of future transmission [38, 39]. In this current study, AGW and JORRP cases for both sexes reached lower incidence levels ten years sooner with the 6-year catch-up than the status quo. Interestingly, although this study focused on female-only 9vHPV vaccinations, total disease cases averted were higher in males than females, mostly due to a greater number of averted AGW cases for males. Routine HPV vaccination coverage of younger females combined with catch-up programs for older female cohorts has previously been shown to lower the incidence of both female and male AGW cases, indicating the effect of herd immunity [22, 38–41]. In this current study, AGW incidence was 68% and 60% lower for the 6-year catch-up scenario than the status quo after 25 years for females and males, respectively. However, AGW incidence returned to similar levels for both sexes after about 70 years, regardless of the length of the catch-up scenarios. JORRP case incidence followed a similar pattern as AGW incidence levels for both sexes, except that JORRP incidence increased back to similar levels after 50 years for both base-case scenarios. This is an indication that although the catch-up extension program would help to considerably decrease the incidence of both AGW and RRP, such extension alone could not completely alleviate these diseases in the Japanese society if lower coverage persists for the routine cohorts. An increase in routine VCR and or alternative vaccination strategies such as universal HPV vaccination would be necessary to eliminate these diseases in Japan within the next century.

This study also evaluated the impact of increasing routine vaccination coverage from 60% to 90% for both the routine vaccination + 3-year and 6-year catch-up scenarios on 9vHPV-related disease cases and deaths. Similar to another modeling study of Japan, increasing routine VCR lowered 9vHPV-related disease incidence faster than extending the catch-up program to six years with no increase in routine VCR [22]. Extending the current catch-up program an additional three years (6-year catch-up) further lowered HPV-related disease incidence compared to the 3-year catch-up status quo regardless of the high routine VCRs tested (60–90%). Although it would be ideal to significantly increase both routine VCR and catch-up VCR, challenges still exist in Japan to increase HPV vaccine uptake. The primary challenge is the remaining vaccine hesitancy and the low cervical cancer screening rates [15, 20, 42]. Despite being a high-income, highly educated country, Japan has one of the lowest global vaccine confidence rates. In general, vaccine-hesitant parents in Japan are more concerned about the perceived risks of the HPV vaccine than HPV vaccine-preventable diseases [20]. Also, a recent survey found that females aged 20–27 were twice as likely to be afraid of the HPV vaccine’s potential side effects if they were unsure about the vaccine or did not want to be vaccinated [43]. This HPV vaccine hesitancy is in stark contrast to Japan’s ability to achieve an 85.5% COVID-19 vaccine acceptance rate five months after approval of the vaccine, resulting in 72.4% complete COVID-19 vaccination coverage of Japan by the end of October 2021 [44]. This coverage level placed it ahead of other high-income countries at a similar time point such as the United Kingdom (67.0%), Germany (66.2%), and the United States (58.6%) [45]. Rapid COVID-19 vaccination coverage of the Japanese population during the pandemic has been attributed to governmental efforts to promote vaccine effectiveness as well as widespread and organized vaccine delivery with multiple public health centers and workplace options available [46, 47]. Therefore, while measures are taken to improve vaccine uptake, short-term solutions of extending the current catch-up program in addition to promoting the increase in routine VCR should be considered to improve the burden of HPV-related diseases and to accelerate cervical cancer elimination in Japan.

Cervical cancer is the one HPV-related disease that can be detected and treated by regular screening of women 20 to 69 years of age [48]. The cervical cancer screening rate in Japan has remained low over the last ten years, ranging from 42.1% to 43.6% from 2013 to 2022, respectively [49]. Previous studies have demonstrated the importance of cervical screening to reduce cervical cancer risk when females are vaccinated after the age of 20 [19, 33, 50]. Females that are not vaccinated prior to this age are more likely to already be infected with the HPV virus, which may lead to a cervical cancer diagnosis 10 to 20 years later [51]. Currently, biennial cytology-based screening is recommended for Japanese women 20 to 69 years of age despite the availability worldwide of more sensitive HPV DNA-based testing [15]. A recent study found that with the low vaccination coverage in Japan, the most cost-effective strategy would be for Japan to switch to HPV DNA-based testing every three years until HPV vaccination coverage in Japan increases back to 70% coverage levels [15]. The results of this current study demonstrate that an extended catch-up vaccination strategy would be a beneficial short-term action for Japan given the current low routine VCR for women.

Analyses from modeling studies are subject to limitations due to assumptions and estimates used for input parameters. For this model, there were assumptions for current and future vaccination as well as cervical cancer screening rates due to the absence of a national database with this information. However, this has occurred for other HPV studies for Japan, and estimates were derived from published public health sources as well as expert opinion [2, 22, 27]. Additionally, the model only considered HPV-related diseases due to HPV types included in the 9vHPV vaccine. Although most disease is caused by the nine HPV types targeted by the vaccine, the total disease case and death estimates in this study may be underestimated. This current study also did not look at the impact of cervical cancer screening rates on HPV-related disease incidence as the objective was to focus only on the impact of extending the catch-up vaccination program. The AGW and JORRP results from this study, showing a remaining level of HPV incidence due to the nine-year gap of routine vaccination coverage, indicate that future analyses should look at the impact of increased gender-neutral routine vaccination rates as well as enhanced cervical cancer screenings on HPV-related disease cases over time. Gender-neutral vaccinations have been shown to be cost-effective measures for several countries, including Japan and the United States, especially when there are low female-only HPV vaccination rates [27, 52]. After the almost nine-year absence of HPV vaccination recommendations, lowering HPV disease transmission in Japan through multiple measures should be considered to help alleviate morbidity and mortality from HPV-related diseases.

##  Conclusions

In conclusion, our analyses demonstrate that routine vaccination in combination with the extension of the current HPV catch-up vaccination program in Japan for an additional three years (2025–2028) would avert an additional 413,454 HPV-related disease cases and 7,184 HPV-related deaths over 100 years. The extended catch-up scenario analyzed in this study indicates that the routine vaccination with the addition of an extended catch-up program would be an effective supplementary strategy that can serve as a supportive, ongoing mechanism to close the remaining immunity gaps and ensure equitable access to HPV vaccination. However, extending the catch-up program provides temporary benefits, thus strengthening and sustaining high routine vaccination coverage should remain the primary public health goal.

## Data Availability

The authors confirm that the data supporting the findings of this study are available within the article.

## Abbreviations

AGW: Anogenital wart
ASR: Age-standardized rate
CIN: Cervical intraepithelial neoplasia
HPV: Human papillomavirus
JAMDAS: Japan Medical Data Survey
JORRP: Juvenile-onset recurrent respiratory papillomatosis
MHLW: Ministry of Health, Labor, and Welfare
NIP: National immunization program
VCR: Vaccine coverage rates
WHO: World Health Organization
2vHPV: Bivalent HPV vaccine
4vHPV: Quadrivalent HPV vaccine
9vHPV: Nonavalent HPV vaccine
